# Human CD8^+^ T Cells from TB Pleurisy Respond to Four Immunodominant Epitopes in Mtb CFP10 Restricted by HLA-B Alleles

**DOI:** 10.1371/journal.pone.0082196

**Published:** 2013-12-12

**Authors:** Li Li, Binyan Yang, Sifei Yu, Xianlan Zhang, Suihua Lao, Changyou Wu

**Affiliations:** 1 Institute of Immunology, Zhongshan School of Medicine; Key Laboratory of Tropical Disease Control Research of Ministry of Education, Sun Yat-sen University, Guangzhou, People's Republic of China; 2 Chest Hospital of Guangzhou, Guangzhou, People's Republic of China; The Ohio State University, United States of America

## Abstract

CD8^+^ T cells are essential for host defense to *Mycobacterium tuberculosis* (Mtb) infection and identification of CD8^+^ T cell epitopes from Mtb is of importance for the development of effective peptide-based diagnostics and vaccines. We previously demonstrated that the secreted 10-KDa culture filtrate protein (CFP10) from Mtb is a potent CD8^+^ T cell antigen but the repertoire and dominance pattern of human CD8 epitopes for CFP10 remained poorly characterized. In the present study, we undertook to define immunodominant CD8 epitopes involved in CFP10 using a panel of CFP10-derived 13–15 amino acid (aa) peptides overlapping by 11 aa. Four peptides in CFP10 were observed to induce significant CD8^+^ T cell responses and we further determined the size of the epitopes involved in each individual peptide tested. Four 9 aa CD8 epitopes were finally identified and deleting a single amino acid from the N or C terminus of either peptide markedly reduced IFN-γ production, suggesting that they are minimum of CD8 epitopes. In the individuals tested, each epitope represented a single immunodominant response in CD8^+^ T cells. The epitope-specific CD8^+^ T cells displayed effector or effector memory phenotypes and could upregulate the expression of CD107a/b upon antigen stimulation. In addition, we found that epitope-specific CD8^+^ T cells shared biased usage of T cell receptor (TCR) variable region of β chain (Vβ) 12, 9, 7.2 or Vβ4 chains. As judged from HLA-typing results and using bioinformatics technology for prediction of MHC binding affinity, we found that the epitope-specific CD8^+^ T cells are all restricted by HLA-B alleles. Our findings suggest that the four epitopes in CFP10 recognized by CD8^+^ T cells might be of importance for the development of Mtb peptide-based vaccines and for improved diagnosis of TB in humans.

## Introduction

Tuberculosis (TB) is one of the leading infectious diseases with eight million new cases and 2–3 million deaths annually worldwide [1]. The only available vaccine, BCG, has limited effectiveness against adult pulmonary TB [2], [3]. Hence, the development of more efficient TB vaccines is urgently needed. Cell-mediated immunity is known to be crucial for protection against TB and most studies have shown that CD4^+^ T cells are essential for protective immunity [4], [5]. However, increasing evidence in animals and humans suggests that CD8^+^ T cells also contribute significantly to immune defenses against TB through lysis of infected cells, production of cytokines and direct microbicidal activity [6]–[12]. Therefore, the most effective vaccine is likely to be one that elicits both CD4^+^ and CD8^+^ T cells responses [12].

Although a number of commonly recognized CD4 Mtb antigens have been described [13], [14], surprisingly little is known about common Mtb antigens recognized by CD8^+^ T cells [15], [16]. Most published evidence indicates that secreted Mtb antigens stimulate protective immunity [17]. 10-KDa culture filtrate protein (CFP10) has been shown to stimulate T cells to produce IFN-γ and exhibit CTL (cytotoxic T lymphocytes) activity in animal models and in humans infected with Mtb, making it excellent candidate for inclusion in an antituberculosis subunit vaccine [18], [19]. In our previous study, we have demonstrated that both CFP10-specific CD4^+^ and CD8^+^ T cells were present in patients with tuberculous pleurisy (TBP) [20]. To date, however, limited CD8^+^ T cell epitopes involved in CFP10 have been identified [21]–[23]. Therefore, the identification of new CTL epitopes is of importance for the analysis of the involvement of CD8^+^ T cells in Mtb infections as well as for vaccine development.

T cell receptor (TCR) is mostly composed of two different polypeptide chains, termed the T-cell receptor α (TCRα) and T-cell receptor β (TCRβ) chain, each chain of which contains one constant (C) region and one variable (V) region. Each Vα (variable region of α chain) or Vβ (variable region of β chain) chain forms three loops that interact with the peptide/MHC molecule. The diversity of the TCR repertoire results from rearrangements of various gene segments, their imprecise joining, the addition of template-independent N nucleotides during this process, and the pairing of different α- and β- chains [24], [25]. TCR Vβ repertoire can indicate which families of T cells are involved in the immune response. Analysis of the TCR Vβ chain distribution is widely used to characterize alterations in the T cell repertoire [26]–[28]. Recent studies about TCR Vβ diversity have been put forward in reports comparing the repertoires of T cell subset. However, limited research has been raised about the TCR Vβ repertoire in *M.tuberculosis* infection [20], [29]. In the present study, we performed flow cytometry analysis, which allows the simultaneous characterization of the Vβ repertoire within antigen-specific T cells as determined by functional analysis.

Using peptides of predicted HLA binding specificity, it is plausible to elicit CD8^+^ T cells capable of recognizing targets. However, one limitation of the peptide-based approaches is that it is difficult to ascertain whether these responses are primed by Mtb infection. Similarly, it remains uncertain as to whether the peptides tested reflect dominant epitopes generated during the course of natural infection.

In the present study, we aimed to determine the immunodominant CD8^+^ T cell epitopes involved in CFP10 in TBP. We used twenty-six overlapping synthetic peptides spanning the CFP10 protein to analyze the magnitude of the CD8 response. We identified and characterized four 9aa peptide of CFP10 that elicited IFN-γ production and CD107a/b expression by CD8^+^ T cells in pleural fluid cells from TBP.

## Materials and Methods

### Ethics statement

Written informed consent was obtained from all patients. Ethics approval for the present study was obtained from the ethics committee of the Zhongshan School of Medicine, Sun Yat-sen University (Guangzhou, China) and the Chest Hospital of Guangzhou (Guangzhou, China).

### Study participants

A total of twenty-seven patients with tuberculous pleurisy (12 females and 15 males, range 19–65 years of age) were recruited from the Chest Hospital of Guangzhou, China. Diagnosis of pleural effusion from TB etiology was based on one of the following criteria: (i) demonstration of MTB on pleural fluid smear (by the Ziehl-Neelsen method); (ii) pleural fluid or pleural biopsy specimens growing *M. tuberculosis* on Lowenstein-Jensen medium; or (iii) histological evidence of caseating granuloma on biopsy specimens of pleural tissue with positive staining for *M. tuberculosis*. Patients who had been previously diagnosed with HIV, HBV, or HCV or with a history of autoimmune diseases were excluded from the study. None of the patients was receiving MTB-related treatments at the time of the sample collection. The study was approved by the Zhongshan School of Medicine Review Board (Guangzhou, China).

### Collection of pleural fluid cells (PFCs) samples and HLA typing

PFCs were isolated by lysing erythrocytes using ammonium chloride solution and resuspended to a final concentration of 2×10^6^ cells/mL in complete RPMI 1640 medium (Invitrogen, Grand Island, NY) supplemented with 10% heat-inactivated fetal calf serum (FCS; HyClone, Logan, UT), 100 U/mL penicillin, 100 µg/mL streptomycin, 2 mM L-glutamine, and 50 µM 2-aacaptoethanol. Low-resolution sequence-based typing of the HLA-A/B/C was performed in Guangzhou Tissue Typing Center, Guangzhou Blood Center (Guangzhou, Guangdong, P.R. China).

### Peptides

In a previous study, we demonstrated that Mtb-specific protein, CFP10, could induce CD4^+^ T cells and CD8^+^ T cells to produce cytokines in PFCs from patients with TBP (20). To identify the exact regions of CFP10 that induces the production of cytokines, we synthesized 26 overlapping 13–15aa peptides that spanned CFP10. Twenty-six 13–15aa peptides that overlapped by 11 aa and spanned the CFP10 protein were synthesized and named C1-C26. Twenty-seven patients with TBP were stimulated with or without a pool of twenty-six mixed CFP10 peptides. In order to further map epitopes, 26 peptides were divided into five pools: CFP10 Pool A, C1 to C5; CFP10 Pool B, C6 to C10; CFP10 Pool C, C11 to C15; CFP10 Pool D, C16 to C20; and CFP10 Pool E, C21 to C26. These pools and single peptide were used in the subsequent experiments. Truncated peptides of 6–9aa were also synthesized, as outlined in the results. All of the peptides were synthesized by Sangon Biotech (Shanghai) Co.,Ltd. Peptide purity was >95%, as assayed by HPLC, and their composition was verified by mass spectrometry. Lyophilized peptides were dissolved at 20 mg/ml in DMSO, aliquoted, and stored at −80°C.

### Reagents

Purified anti-CD28 (clone CD28.2) and anti-CD49d (clone 9F10) mAbs were purchased from BD Biosciences (San Jose, CA). The following mAbs were used for phenotypic and intracellular cytokine analysis and were purchased from BD Biosciences (San Jose, CA, USA): CD4-APC-Cy7 (SK3), CD62L-PE (Dreg56), CCR7-PE (3D12), CD45RO-FITC (UCHL1), CD8-eFluor450 (RPA-T8), CD8-peridinin chlorophyll protein (CD8-PerCP) (clone SK1), TNF-α-PE-Cy7 (MAb11), CD127-APC (hIL-7R-M21), IFN-γ-APC (clone B27) and isotype-matched control antibodies. CD27-APC (O323) was obtained from Biolegend (San Diego, CA). CD3-PE-TR (Clone S4.1) was purchased from Invitrogen (Carlsbad, CA). IFN-γ-FITC (45.15) was purchased from Beckman Coulter (Fullerton, CA).

### Flow cytometry

For the detection of intracellular cytokines, PFCs were incubated at a concentration of 2×10^6^ cells/mL with 1 µg/mL peptides plus 1 µg/mL anti-CD28 and 1 µg/mL anti-CD49d for 8 h in the presence of brefeldin A (BFA, 10 µg/mL; Sigma-Aldrich, St Louis, MO). For the medium control, the cells were not treated with either anti-CD28 or anti-CD49d. For the detection of CD107a/b, PFCs were stimulated with the indicated CFP10 peptides plus CD107a-FITC and CD107b-FITC. One hour later, Brefeldin A and Monensin (1 µL/mL, BD Biosciences Pharmingen) were added and the plates were incubated for another 5 hours. After stimulation, cells were washed with PBS containing 0.1% BSA and 0.05% sodium azide. Cells were incubated with antibodies for surface staining and then fixed with 4% paraformaldehyde, permeabilized with PBS containing 0.1% saponin and stained for intracellular cytokines. Flow cytometry was performed using BD FACS Calibur cytometer (BD Biosciences) or FACSAria II (BD Biosciences) and the data were analyzed using FlowJo software (TreeStar, San Carlos, CA, USA).

### TCR Vβ repertoire analyzing

PFCc were stimulated with the indicated peptides. After stimulation, cells were stained with CD3-PE-TR, CD4-APCcy7, CD8-PerCP and the IOTest Beta Mark TCR Vβ repertoire kit (Beckman Coulter, Inc., Brea, CA) which allowed staining of 24 TCR Vβ chains. Cells were then fixed with 4% paraformaldehyde, permeabilized with PBS containing 0.1% saponin and stained with IFN-γ-APC and TNF-α-PEcy7. Flow cytometry was performed using a BD FACS Aria II. Lymphocytes were first gated according to FSC/SSC parameters, then by selection of CD3^+^CD8^+^ T cells. The IOTest Beta Mark TCR Vβ Repertoire Kit contains 8 vials (A to H) of FITC- and PE-conjugated TCR Vβ antibodies. Each vial contains a mixture of 3 TCR Vβ antibodies, corresponding to a total of 24 different specificities. The 24 mAbs are as follows: Vβ1 (BL37.2), Vβ2 (MPB2D5), Vβ3 (CH92), Vβ4 (WJF24), Vβ5.1 (IMMU157), Vβ5.2 (36213), Vβ5.3 (3D11), Vβ7.1 (ZOE), Vβ7.2 (ZIZOU4), Vβ8 (56C5.2), Vβ9 (FIN9), Vβ11 (C21), Vβ12 (VER2.32), Vβ13.1 (IMMU222), Vβ13.2 (H132), Vβ13.6 (JU74.3), Vβ14 (CAS1.1.3), Vβ16 (TAMAYA1.2), Vβ17 (E17.5F3), Vβ18 (BA62.6), Vβ20 (ELL1.4), Vβ21.3 (IG125), Vβ22 (IMMU546), Vβ23 (AF23).

### Statistical analysis

Wilcoxon matched pairs test (Two-tailed) was used to determine the statistical differences between the groups using GraphPad Prism software version 5. A value of *p*<0.05 was considered statistically significant.

## Results

### CFP10 peptides induce the production of IFN-γ and TNF-α by both CD8^+^ and CD4^+^ T cells from pleural fluid cells (PFCs) by patients with tuberculous pleurisy (TBP)

**Figure 1:**
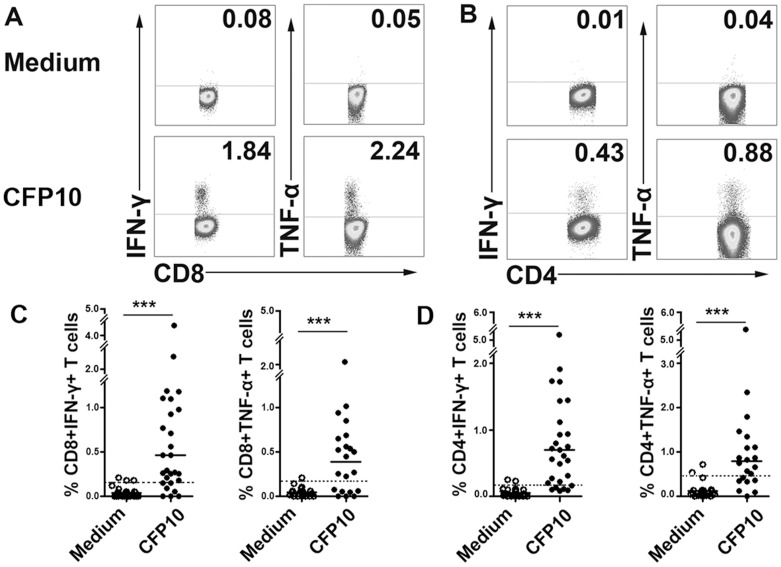
The production of IFN-γ and TNF-α by both CD8^+^ and CD4^+^ T cells from pleural fluid cells (PFCs) by patients with tuberculous pleurisy (TBP) in response to 13-15aa culture filtrate protein 10 (CFP10) peptides. (A, B) PFCs from patients with TBP were cultured with or without a mixture of twenty-six CFP10 peptides for 8 h and stained with anti-CD4, CD8, IFN-γ and TNF-α mAbs. The expression of IFN-γ and TNF-α by CD8^+^ (A) and CD4^+^ (B) T cells was analyzed. Numbers in quadrants indicate percentages of the cells in each population. (C, D) Statistical results of IFN-γ and TNF-α expression by CD8^+^ (C) and CD4^+^ (D) T cells from twenty-seven patients with TBP. Open circles indicate cultures without CFP-10 and filled circles indicate cultures with CFP-10 peptides. Horizontal bars indicate the medians for the indicated conditions. ***, P<0.001. Dashed line represents the cutoff of IFN-γ or TNF-α-positive CD8^+^ or CD4^+^ T cells.

As shown in Fig. 1A and B, mixed CFP10 peptides elicited considerable levels of IFN-γ and TNF-α production by both CD8^+^ and CD4^+^ T cells. For the twenty-seven patients tested, CFP10 peptides induced IFN-γ production by CD8^+^ T cells from 20 patients (mean ± SD, 0.91±1.01% IFN-γ^+^ cells) and by CD4^+^ T cells from 21 patients (1.08±1.01% IFN-γ^+^ cells). Similarly, the results showed that CFP10 peptides induced TNF-α production by CD8^+^ T cells from 14 patients (mean ± SD, 0.68±0.47% TNF-α^+^ cells) and by CD4^+^ T cells from 13 patients (1.49±1.35% TNF-α^+^ cells) in twenty-one patients. When the cutoff was set at mean+2SD of cultures without stimulation, CFP10 were recognized by 74.07% (IFN-γ) and 66.67% (TNF-α) of responders for CD8^+^ T cells, 77.78% (IFN-γ) and 61.90% (TNF-α) of responders for CD4^+^ T cells (Fig 1C and D). The major concerns might be the apparent diversity and polymorphism of the MHC molecules and the affinity to antigenic epitopes, which would lead to distinct reactivity to the same antigen. In addition, the functional diversities of antigen-specific T cells could also accout for the distinct response in each individual.

### Specific CD8^+^ T cell responses to individual pooled peptides of CFP10 in patients with TBP

**Figure 2:**
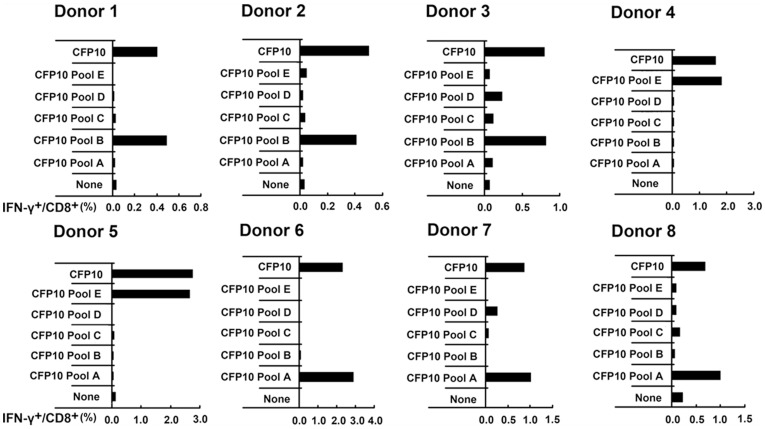
The expression of IFN-γ by CD8^+^ T cells in PFCs from patients with TBP in response to pooled peptides of 13–15aa CFP10. Twenty-six synthetic peptides that spanned the entire CFP10 sequence were divided into five pools (each pool contains 5–6 peptides): CFP10 Pool A, CFP10 Pool B, CFP10 Pool C, CFP10 Pool D, CFP10 Pool E and CFP10 total peptides, as described in Materials and methods. PFCs from eight CFP10-responsive patients were unstimulated or stimulated with total CFP10 peptides or five pools of CFP10 peptides for 8 h. The expression of IFN-γ by CD8^+^ T cells was assessed by flow cytometry. The production of IFN-γ by CD8^+^ T cells for the indicated conditions from eight independent patients was shown.

**Figure 3:**
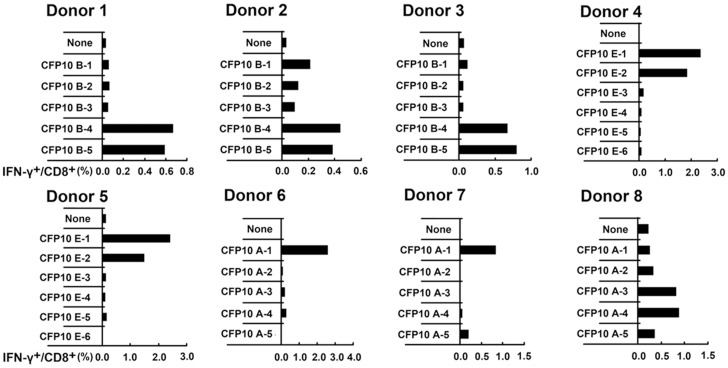
The expression of IFN-γ by CD8^+^ T cells in PFCs from patients with TBP in response to individual peptide of 13–15aa CFP10. PFCs from eight CFP10-responsive patients were unstimulated or stimulated with individual 13–15aa CFP10 peptide that together constituted the positively selected CFP10 pools. The expression of IFN-γ by CD8^+^ T cells for the indicated conditions was shown for each patient.

To identify the peptides involved in CFP10 that were recognized by CD8^+^ T cells, we divided mixed twenty-six peptides into five pools (each pool contains 5–6 peptides): CFP10 Pool A, CFP10 Pool B, CFP10 Pool C, CFP10 Pool D and CFP10 Pool E. PFCs from eight patients with strong CFP10 directed CD8^+^ T cell response were selected and stimulated with each CFP10 pool respectively, and the frequency of IFN-γ^+^CD8^+^ T cells was measured by FACS. The mixed CFP10 peptides elicited IFN-γ production by CD8^+^ T cells from all eight patients. As demonstrated in Fig. 2, of these eight individuals, strong CD8^+^ T cell responses were observed against CFP10 Pool B for donor1, 2 and 3, CFP10 Pool E for donor 4 and 5 while CFP10 Pool A for donor 6, 7 and 8. In addition, the frequencies of CD8^+^ T cells specific for the peptide pool and mixed CFP10 peptides were remarkably concordant. To further determine which single peptide was responsible for the respective response elicited by the CFP10 pool, each possible 13–15aa peptide that comprised the CFP10 pool was then tested for reactivity. Interestingly, CFP10 B-4 and CFP10 B-5 elicited the CD8^+^ T cells responses for both donor 1, 2 and 3. CFP10 E-1 and CFP10 E-2 stimulated the CD8^+^ T cell responses for both donor 4 and 5. CFP10 A-1 elicited the CD8^+^ T cell responses for both donor 6 and 7. CFP10 A-3 and CFP10 A-4 elicited the CD8^+^ T cell response for donor 8 (Fig. 3).

### The minimal epitope recognized by CFP10-specific CD8^+^ T cells

**Figure 4:**
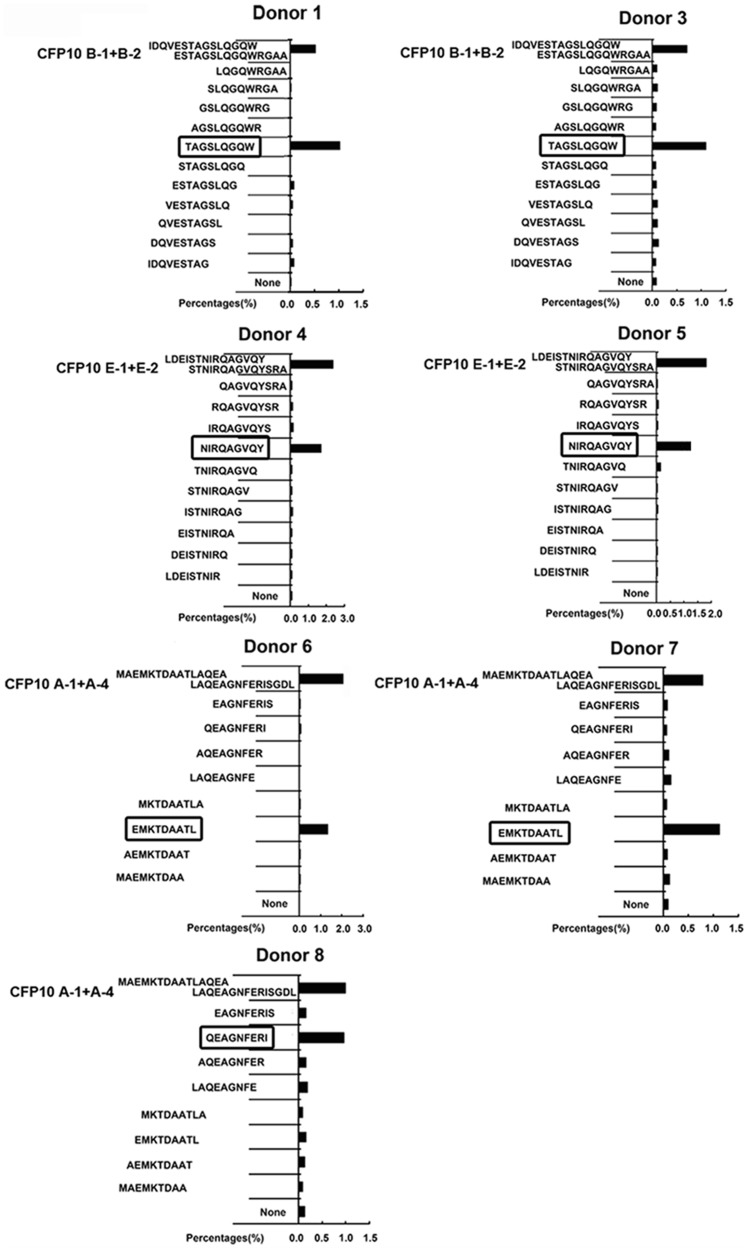
The production of IFN-γ by CD8^+^ T cells in PFCs from patients with TBP in response to 9aa peptide within CFP10. The production of IFN-γ by CD8^+^ T cells in response to a panel of truncated 9aa peptides from the positively selected 13–15aa CFP10 peptide was tested. PFCs from seven CFP10-responsive patients were unstimulated or stimulated with 9aa peptides. The expression of IFN-γ by CD8^+^ T cells for the indicated conditions was analyzed and shown for each patient.

**Figure 5:**
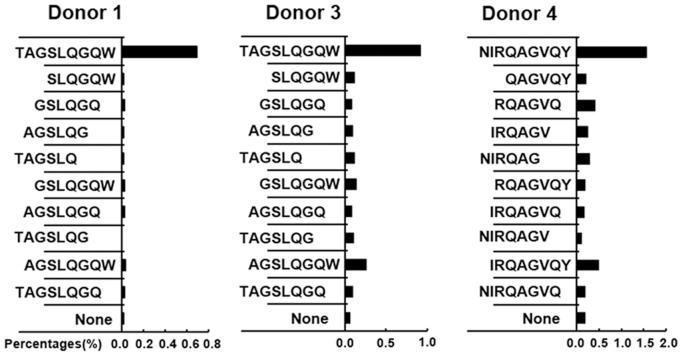
Minimal epitopes within CFP10 recognized by CD8^+^ T cells in PFCs from patients with TBP. A panel of truncated (6aa, 7aa or 8aa) peptides was synthesized to further map the minimal epitopes within CFP10 recognized by CD8^+^ T cells. PFCs from three CFP10-responsive patients were unstimulated or stimulated with truncated peptides. The expression of IFN-γ by CD8^+^ T cells for the indicated conditions was analyzed and shown for each patient.

In order to identify the minimal peptide recognized by CD8^+^ T cells involved in CFP10, each possible 9aa peptide from the 13–15aa peptide was then synthesized and tested for reactivity. PFCs from seven CFP10- responsive donors were further stimulated with each 9aa peptide, and the frequencies of IFN-γ^+^CD8^+^ T cells were measured by FACS. Positive reactivity towards peptides was confirmed at least twice in the same donor. For donor 1 and 3, the 9aa peptide TAGSLQGQW (CFP10_35–43_) elicited comparable frequency of IFN-γ^+^CD8^+^ T cells to that induced by CFP10 B-4 and CFP10 B-5. For donor 4 and 5, the 9aa peptide NIRQAGVQY (CFP10_75–83_) yielded similar frequencies of IFN-γ^+^CD8^+^ T cells as the CFP10 E-1 and CFP10 E-2. For donor 6 and 7, the 9aa peptide EMKTDAATL (CFP10_3–11_) elicited comparable frequencies of IFN-γ^+^CD8^+^ T cells as CFP10 A-1. For donor 8, the 9aa peptide QEAGNFERI (CFP10_13–21_) yielded similar frequency of IFN-γ^+^CD8^+^ T cells as CFP10 A-3 and CFP10 A-4 (Fig. 4). Moreover, the frequencies of CD8^+^ T cells responding to the 9aa peptide and the 13–15aa peptide containing the 9aa epitope were remarkably concordant. To determine whether the above-mentioned four 9aa peptides contain minimal epitopes, we deleted 1–3 amino acids from either the N or C terminus of these peptides. Interestingly, removal of a single amino acid from either end of both peptides remarkably reduced IFN-γ production by CD8^+^ T cells (Fig. 5). Our results demonstrated clearly that the minimal epitope for these observed peptides were 9aa in length.

### TCR Vβ repertoire analysis of CFP10 epitope specific CD8^+^ T cells

**Figure 6:**
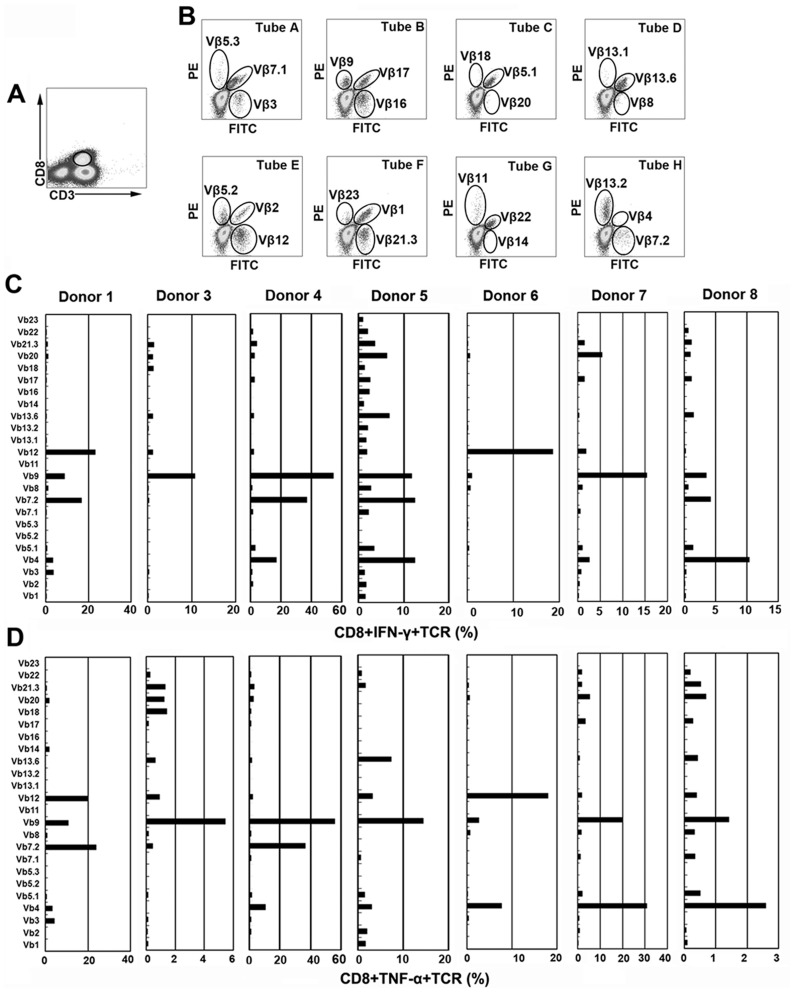
TCR Vβ repertoires of CFP10 9aa peptide-specific IFN-γ or TNF-α-producing CD8^+^ T cells in PFCs from TBP. (A) Representative gating strategy of CD8^+^ T cells as assessed by flow cytometry in PFCs was shown. CD8^+^ T cells were gated according to the expression of CD3 and CD8. (B) The expression of 24 TCR Vβ chains by CD8^+^ T cells was assessed. Analysis of 24 TCR Vβ chains was performed by staining in eight tubes, from A to H. In each tube, one TCR Vβ antibody was conjugated to FITC, another to PE, and a third to both FITC and PE. (C) PFCs were stimulated with each 9aa peptide (Donor 1 and Donor 3: TAGSLQGQW; Donor 4 and Donor 5: NIRQAGVQY; Donor 6 and Donor 7: EMKTDAATL; Donor 8: QEAGNFERI) for 8 h. Percentages of IFN-γ production by CD8^+^ T cells expressing each TCR Vβ chain was indicated for each donor. (D) Percentages of TNF-α production by CD8^+^ T cells expressing each TCR Vβ chain was indicated for each donor.

To precisely define CFP10 epitope specific CD8^+^ T cells from PFCs, we further characterized TCR Vβ repertoire of CFP10 epitope specific CD8^+^ T cells on the basis of IFN-γ and TNF-α production. CD3^+^CD8^+^ T cells were first gated (Fig. 6A), the expression of each TCR Vβ was further demonstrated (Fig. 6B). Thereafter, the production of IFN-γ and TNF-α within each TCR Vβ subfamily was analyzed. Of the seven patients analyzed, two patients (Donor 1 and 6) had the highest proportion of IFN-γ production by TCR Vβ12, three patients (Donor 3, 4 and 7) by TCR Vβ9 and the other two patients (Donor 5 and 8) by TCR Vβ4 (Fig. 6C). To further confirm the results, we analyzed the production of TNF-α within these TCR Vβ subfamilies as well. Interestingly, the results were remarkably similar as that for IFN-γ. Three patients (Donor 3, 4 and 5) had the highest proportion of TNF-α production by TCR Vβ9, two patients by TCR Vβ 4 (Donor 7 and 8) and the other two patients by TCR Vβ7.2 (Donor 1) or Vβ12 (Donor 6), respectively (Fig. 6D). Our results indicated that CFP-10 epitope specific cytokine-producing CD8^+^ T cells had distinct TCR Vβ repertoire usage for different patients, which was probably due to the different types of HLA molecules. However, some TCR Vβ families were much more frequently used, such as TCR Vβ12, Vβ9, Vβ7.2 or Vβ4, compared with other TCR Vβ families, suggesting their special role in the CD8^+^ T cell responses at the local site of Mtb infection.

### CFP10 epitope specific CD8^+^ T cells displayed effector or effector memory phenotype

**Figure 7:**
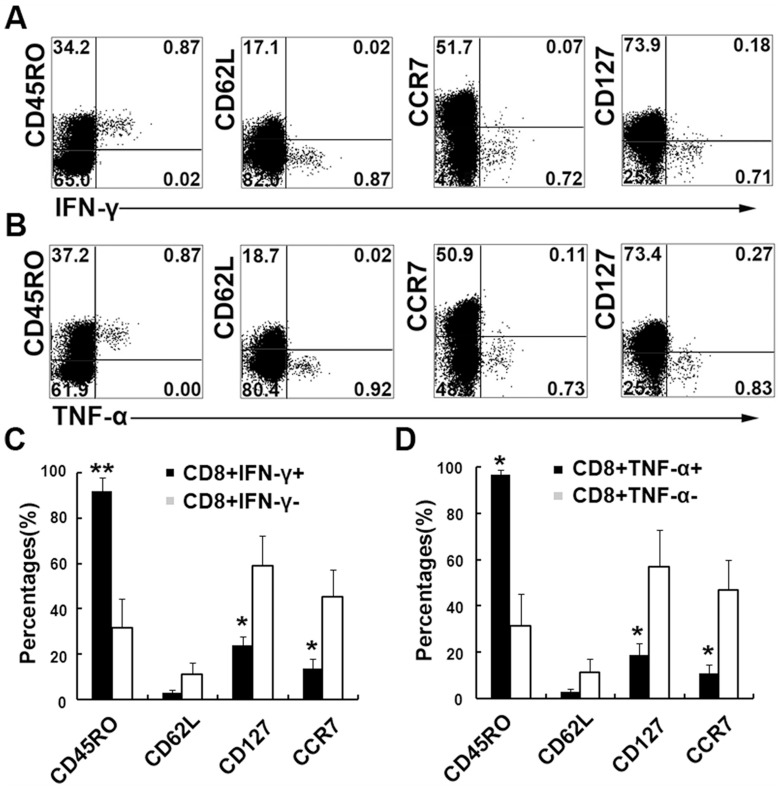
Memory CD8^+^ T cells in PFCs from TBP in response to 9aa CFP10 peptides for the production of IFN-γ or TNF-α. PFCs from CFP10 peptide-responsive patients were stimulated with the indicated 9aa CFP10 peptide for 8 h. (A, B) The expression of CD45RO, CD62L, CCR7, CD127 and IFN-γ (A) or TNF-α (B) by CD8^+^ T cells was assessed by flow cytometry. Numbers in quadrants indicate percentages of the cells in each population. (C, D) Statistical results of CD45RO, CD62L, CCR7 and CD127 expression by CD8^+^IFN-γ^+^/CD8^+^IFN-γ^−^ cells (C) and by CD8^+^TNF-α^+^/CD8^+^ TNF-α^−^ cells (D). Data shown are results from three independent experiments. *, P<0.05; **, P<0.01.

We further detected several surface markers to evaluate the phenotype of CFP epitope specific CD8^+^ T cells on the basis of IFN-γ or TNF-α production. The results clearly indicated that CFP10 epitope specific IFN-γ-producing CD8^+^ T cells were effector or effector memory T cells with the phenotype of CD45RO^+^CD62L^−^CCR7^−^CD127^−^ (Fig. 7A). We also evaluated the phenotype of TNF-α-producing CD8^+^ T cells following stimulation with the 9aa peptide. The results were similar to those for IFN-γ production with the expression of CD45RO^+^CD62L^−^CCR7^−^CD127^−^ (Fig. 7B). Statistic results showed that both IFN-γ^+^CD8^+^ and TNF-α^+^CD8^+^ T cell subsets expressed significantly higher levels of CD45RO and lower levels of CCR7 and CD127 compared with IFN-γ^−^CD8^+^ and TNF-α^−^CD8^+^ T cells. Taken together, CFP10 epitope specific CD8^+^ T cells displayed effector or effector memory phenotype and might thus contribute to immediate effector functions in response to Mtb infection at local sites.

### CFP10 epitopes induced coexpression of CD107a/b and IFN-γ or TNF-α

**Figure 8:**
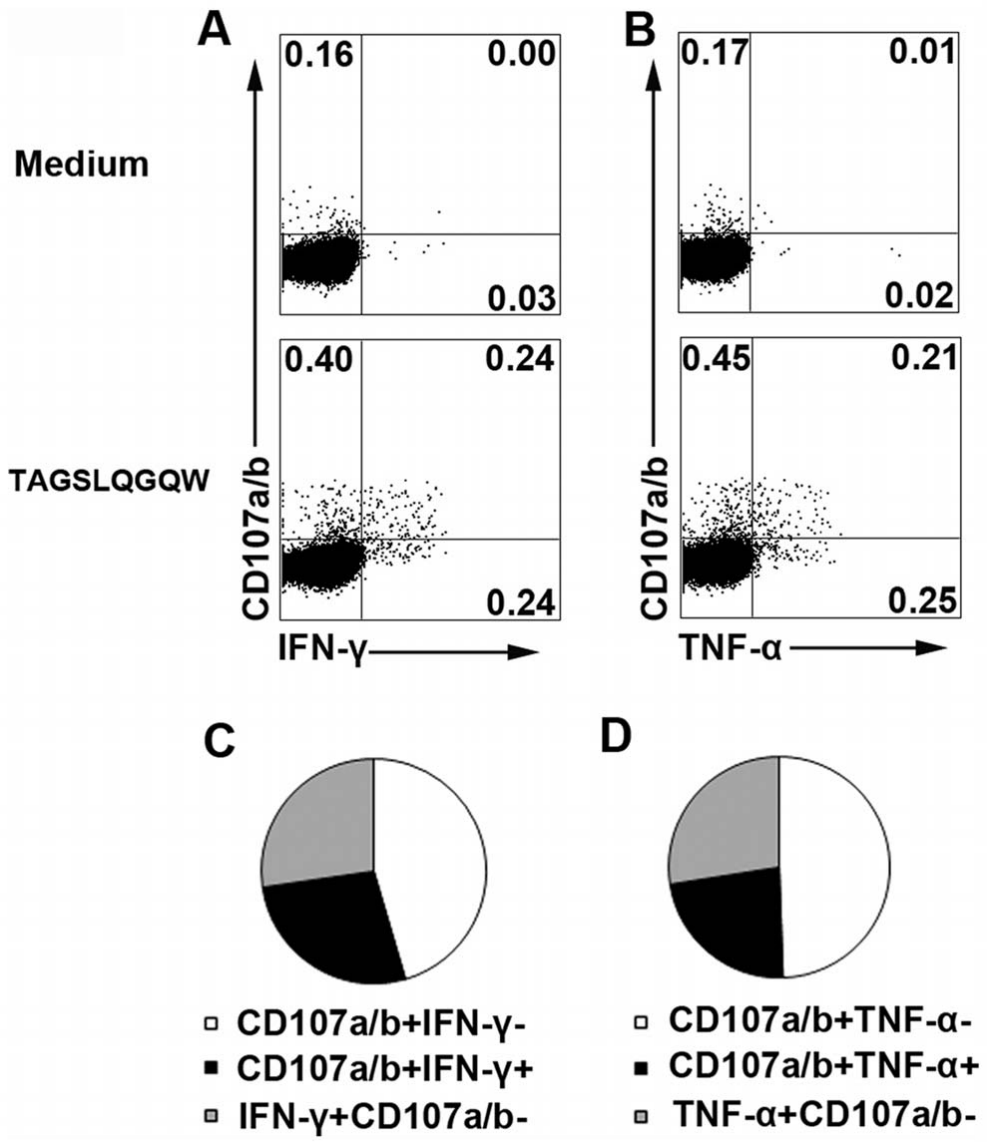
Coexpression of CD107a/b and IFN-γ or TNF-α in CD8^+^ T cells following stimulation with 9aa CFP10 peptide. PFCs were stimulated with or without TAGSLQGQW (CFP10_35–43_) in the presence of anti-CD107a/b mAbs for 8 h. (A, B) The expression of CD107a/b, IFN-γ (A) and TNF-α (B) by CD8^+^ T cells was assessed by flow cytometry. Numbers in quadrants indicate percentages of the cells in each population. (C, D) Coexpression of CD107a/b and IFN-γ (C) or TNF-α (D) by CD8^+^ T cells was shown. The data were quantified and presented in a pie chart, in which each slice of the pie represents the fraction of the median frequencies of a given quadrant. Data shown are representative of three independent experiments with similar results.

To further determine whether the CFP10 epitope specific CD8^+^ T cells have cytolytic potential, we examined the expression of CD107a/b and assessed its correlation with the production of IFN-γ or TNF-α. The results showed that, without any stimulation, neither IFN-γ nor TNF-α were produced by CD8^+^ T cells. However, low levels of CD107a/b were detected by CD8^+^ T cells (Fig. 8A and B). Importantly, significantly higher levels of both CD107a/b and cytokines were induced upon activation with the 9aa peptide (Fig. 8A and B). In addition, we found almost half of the cytokine-producing CD8^+^ cells coexpressed CD107a/b (Fig. 8C and D). Taken together, CFP-10 epitope specific CD8^+^ T cells might have cytotoxic function at the local site of Mtb infection.

### HLA-Ityping and HLA binding affinity to CD8^+^ T cell epitopes

Low-resolution sequence-based typing of the HLA-A/B/C loci in the seven donors tested was summarized (Table 1). Interestingly, the donors who reacted to the same epitope also shared one HLA-B allele. Donor 1 and 3 shared HLA*B58 and Donor 4, 5, 6 and 7 shared HLA*B15. To more definitively identify the restriction alleles for these CD8^+^ T cell epitopes, we predicted HLA binding affinity of the HLA-Iallele to the CD8^+^ T cell epitopes that we identified. HLA-binding scores were counted by peptide-binding prediction method, artificial neural network (ANN), which was available for public use in the Immune Epitope Database (http://www.immuneepitope.org). Prediction values are given in IC50 values, which represent the equilibrium dissociation constant (KD) of the peptide in relation to a particular HLA molecule. The lower the value of IC_50_ is the stronger the binding affinity. The IC_50_ for each epitope was determined against a panel of human HLA molecules. Importantly, the HLA allele that was predicted to have the highest binding affinity to the indicated epitope was consistent with the HLA typing results of the donor tested. For donor 1 and 3, the epitope TAGSLQGQW (CFP10_35–43_) was predicted to have the highest binding affinity to HLA-B*5801 with the IC_50_ of 24.99, which was consistent with their HLA typing results (shared HLA*B58), suggesting that the epitope CFP10_35–43_ was restricted by HLA-B*5801. Similarly, for donor 4 and 5, the epitope NIRQAGVQY (CFP10_75–83_) was predicted to have the highest binding affinity to HLA-B*1502 (IC_50_:108.77) and HLA-B*1501 (IC_50_:142.91), which was also consistent with their HLA typing results (shared HLA*B15), suggesting that the epitope CFP10_75–83_ was restricted by HLA-B*1502 or HLA-B*1501. For donor 6 and 7, the epitope EMKTDAATL (CFP10_3–11_) was predicted to have the highest binding affinity to HLA-B*0801 (IC_50_: 394.88) and HLA-B*1503 (IC_50_: 700.23). As further judged from their HLA typing results (shared HLA*B15), the epitope CFP10_3–11_ should be restricted by HLA-B*1503. For donor 8, the epitope QEAGNFERI (CFP10_13–21_) was predicted to have the highest binding affinity to HLA-B*4001 (IC_50_: 32.06), which happened to be consistent with the HLA typing results of HLA-B*40, suggesting that the epitope CFP10_13–21_ was restricted by HLA-B*4001 (Table 2). We could thus identify the HLA allele that binds to the epitope indicated. Our data demonstrate that the CD8^+^ T cell epitopes bound avidly to HLA allele, and show a high degree of concordance between the T cell epitope data and HLA binding data.

**Table 1:** Overview of sequence-based HLA-Ityping of the donors tested.

Donor	Sex	Age	HLA-A	HLA-B	HLA-C
1	M	52	A*33,-	B*58,-	C*03,-
3	F	24	A*02,-	B*58,*51	C*03,*14
4	F	30	A*02,*24	B*15,*46	C*01,*08
5	F	19	A*11,-	B*15,-	C*01,*08
6	M	26	A*11,*24	B*15,*40	C*03,*07
7	F	58	A*11,*31	B*15,*13	C*07,-
8	M	57	A*02,*11	B*40,-	C*03,*14

**Table 2:** Summary of CD8^+^ T cell epitopes and their MHC binding affinity.

Donor	Epitope Location	Epitope Sequence	HLA allele	MHC binding affinity (IC_50_ nM)
1	35–43	TAGSLQGQW	B*5801	24.99
3	35–43	TAGSLQGQW	B*5801	24.99
4	75–83	NIRQAGVQY	B*1502, B*1501	108.77, 142.91
5	75–83	NIRQAGVQY	B*1502, B*1501	108.77, 142.91
6	3–11	EMKTDAATL	B*1503	700.23
7	3–11	EMKTDAATL	B*1503	700.23
8	13–21	QEAGNFERI	B*4001	32.06

## Discussion

The aim of the present study was to identify CD8^+^ T cell epitopes involved in CFP10. CFP10 and ESAT-6 are part of an operon that is deleted from BCG [30], [31]. We have previously demonstrated that CFP10 protein could induce both CD4^+^ and CD8^+^ T cell immunity [20]. Therefore, CFP10 may be a candidate for inclusion in a tuberculosis vaccine designed to elicit both CD4^+^ and CD8^+^ T cell response. Importantly, because the protein is not present in BCG, it may serve to supplement or boost immunity in persons who have previously received the BCG vaccine. In the present study, we synthesized twenty-six 13–15aa peptides that spanned the whole sequence of CFP10. Initially, in order to ensure the specificity of the response induced by CFP10 peptides, we tested the response of PFCs to non relevant synthetic peptide and also the response of cells from normal donors to the CFP10 peptides. We found that non relevant synthetic peptide could not induce IFN-γ production and that no response was induced in normal donors as well. Using PFCs from patients with TBP, we have identified four 9aa CD8 epitopes, CFP10_35–43_, CFP10_75–83_, CFP10_3–11_ and CFP10_13–21_. Consistent with previous work, CFP10_75–83_ and CFP10_3–11_ have been identified as the CD8^+^ T cell epitopes by using CD8^+^ T cell clones generated from Mtb-infected DCs [23]. Previous work has also shown that CFP10_85–94_ and CFP10_2–11_ (10aa in length) are CD8 epitopes by using human CD8^+^ T cell clones [22]. In addition to the epitopes mentioned above, CFP10_2–9_ (AEMKTDAA), CFP10_2–12_ (AEMKTDAATLA) and CFP10_49–58_ (TAAQAAVVRF) were also identified as CD8^+^ T cell epitopes involved within CFP10 [23]. In our study, however, we found that the minimal epitopes were 9aa in length and that deletion of a single amino acid from either the N or C terminus of these peptides remarkably reduced IFN-γ production by CD8^+^ T cells.

Regarding the phenotype of epitope-specific CD8^+^ T cells, our data indicated that either IFN-γ or TNF-α-producing CD8^+^ T cells were effector or effector memory T cells with the phenotype of CD45RO^+^CD62L^−^CCR7^−^CD127^−^, indicating that these cells might contribute to immediate effector functions in response to Mtb infection at local sites. In addition to cytokine production profiles, cytolytic capacity is generally considered the most essential ability for CD8^+^ T cells. CD107a and CD107b are intracellular proteins that are structural components of cytotoxic granules. Transient CD107a/b expression on the surface of lymphocytes could reflect the cytotoxic capacity of these cells [32]. Our data showed that CD8^+^ T cells upregulated CD017a/b expression upon the epitope stimulation, indicating that these CD8^+^ T cells have potential cytolytic ability. Moreover, coexpression of either IFN-γ or TNF-α and CD107a/b were observed, which might contribute to the optimal biological functions of CD8^+^ T cells.

We also analyzed the TCRVβ repertoire of these epitope-specific CD8^+^ T cells. We combined detection of IFN-γ or TNF-α production with TCRVβ staining and analyzed the major TCRVβ chains of epitope-specific CD8^+^ T cells. Although distinct TCRVβ repertoire was observed for different individuals, significant biased usage of TCRVβ12, 9, 7.2 and 4 were found in all persons detected, suggesting their special role in the CD8^+^ T cell responses at the local site of Mtb infection.

In order to further clarify the HLA restriction alleles for four CD8 epitopes, we performed HLA-I typing and prediction of HLA binding affinity of the HLA-I allele to the epitopes that we have identified. Our data indicated that all of the four epitopes are restricted by HLA-B molecules. CFP10_35–43_ is restricted by HLA-B*5801, CFP10_75–83_ by HLA-B*1502, CFP10_3–11_ by HLA-B*1503 and CFP10_13–21_ by HLA-B*4001. Previous work has shown that CFP10_75–83_ and CFP10_3–11_ are HLA-B*1502 and HLA-B*0801 restricted, respectively, for human CD8^+^ T cell clones [21]. The possible reason for this difference is the biased isolation of T cell clones. Our data that all of the four epitopes are HLA-B restricted are consistent with those reported by Lewinsohn et al, in which all but one of the epitopes that have been mapped in Mtb is restricted by HLA-B molecules [23]. The possible reason may be that Mtb antigens preferentially bind to the HLA-B molecules or interfere with HLA-A processing and presentation. In TB, evaluation of CD8 epitopes based solely on HLA binding affinity has always focused on HLA-A2 [33]-[39], and has often failed to define dominant epitopes. Our results and previous study clearly indicated that future work should focus on HLA-B alleles when defining TB epitopes. Our work also demonstrated that all of the minimal epitopes that are identified exhibit high binding affinity to the HLA allele that is expressed by the cognate individual, suggesting substantial accuracy and reliability of HLA prediction method.

In conclusion, we have identified four 9aa CFP10 CD8 epitopes that appear to be restricted by HLA-B molecules. These results may have important implications for a new design of epitope-based TB diagnosis and candidate TB vaccine.
